# Patients' Consent to Operations

**Published:** 1896-11-21

**Authors:** 


### Patients' Consent to Operations.

While congratulating Dr. Cullingworth on the verdict in the case of Beatty v. Cullingworth, we must also congratulate the medical profession upon the terms in which the learned judge summed up the case to the jury. It is now more than four years since the operation was performed which was the basis of this action, and since then the case has come before the Courts in various forms.

The grievance seems to have been the removal of both the ovaries, instead of restricting the operation to the removal of that one alone which was recognised beforehand to be diseased, the plaintiff claiming damages for injury to her from the defendant's malpractice in performing an operation wrongfully and without her consent. The defendant, however, alleged that the plaintiff had left the operation in his hands, and that what he did was necessary.

Two of the main points raised in the case were, then, whether the operation, as performed by Dr. Cullingworth, was necessary, and whether it was expressly forbidden by the plaintiff. With regard to the first of these points much need not be said. The fact that the actual condition of the parts is often not ascertainable until they are laid bare in the cou se of the operation, much invalidates the evidence of those who were not present at the time. As to consent, it is quite clear that the patient did entertain a great objection to the idea of losing both her ovaries. But it is also clear from the evidence of Dr. Cullingworth that he did not in any way bind himself to remove only one. He is reported to have said that on the day before the operation the plaintiff said she could not consent to run any risk of having both ovaries removed. Witness replied that there was strong reason for thinking that the question would not arise, but that no one could be absolutely sure till the operation was proceeding. Witness added that he could not bind himself by any promise. "He would not have undertaken the operation unless he had understood he was unfettered." He also said "After the patient came into the room, she said, 'Dr. Cullingworth, if you find both ovaries diseased you must remove neither;' to which he replied, 'You really must leave that to me, nurse. I know your wishes. You may be sure I shall not remove anything that I can help.' The plaintiff made no reply, and got on to the operating-table, and took the anæsthetic. Had she raised any objection to his statement he would not have proceeded with the operation." We have quoted this evidence because it almost exactlv represents what happens scores of times in the professional life of every surgeon. On the one hand, we have a patient anxious to obtain guarantees that the operation shall not go beyond a certain point; on the other, a surgeon anxious to do his best for his patient and refusing to be fettered. A patient finally giving consent, and yet at the last moment again raising the old point, and, on being assured that the surgeon must be left a free hand, making no response; a surgeon with the actual nature of the case at last laid bare before him, and with problems of life and death hanging on the correctness of his decision, acting on the consent given rather than on the protest raised but unpersisted in.

Just this state of affairs happens again and again in every hospital, and it is out of the question that when consent has been given a patient can be allowed to risk her life by gasping out at the last moment some foolish limitation by which the success of the operation may be imperilled.

It is in regard to this that we have to thank the judge for his summing up. He is reported to have said that, if a medical man undertook an operation, "he should have thought it was a humane thing for him to do everything in his power to remove the mischief, provided that he had no absolute definite instructions not to operate."

In view of the fact that, according to Dr. Cullingworth's own evidence, the plaintiff did at the last moment raise a protest, and that in the end he only obtained consent by silence, the summing up and the verdict given in this case are, we venture to think, matters of no small importance in regard to a constantly recurring practical difficulty. We must not, however, be misunderstood. It is clear that a patient has a perfect right to impose limits on an operation in such a way that no surgeon will be justified in exceeding them; but they must be "absolute definite instructions." At the same time we may express the opinion that only very rarely can it be wise or even justifiable for a surgeon to undertake to do an operation under such conditions.

